# Supplementary material for: Repurposing the selective estrogen receptor modulator bazedoxifene to suppress gastrointestinal cancer growth
Source: EMBO Mol Med. 2019 Mar 18;11(4):e9539. doi: 10.15252/emmm.201809539 (PMC6460354; doi:10.15252/emmm.201809539)
Supplement: Supplementary file 1 — Appendix [file EMMM-11-e9539-s001.pdf]

Repurposing the selective estrogen receptor modulator *bazedoxifene* to suppress gastrointestinal cancer growth

Appendix

Table of contents

Appendix Figure Legends

Appendix Figure S1: SERM-selective of suppression of IL11-mediated STAT3 signaling activity

Appendix Figure S2: Models of *bazedoxifene* interactions with Site III residues of the hexameric gp130 complex.

Appendix Figure S3: Identification of tumor-associated immune infiltrates by flow cytometry

Appendix Figure S4: Tumor infiltrating T-cells populations are unaffected in *bazedoxifene* (BZA) or vehicle-treated *Cdx2*$^{\mathrm{CreERT2}}$;*Apc*^{flox} mice

Appendix Figure S5: Tumor infiltrating myeloid and B cell populations are unaffected in *bazedoxifene* (BZA) or vehicle-treated *Cdx2*$^{\mathrm{CreERT2}}$;*Apc*^{flox} mice.

Appendix Table S1: QPCR Primer Sequences

Appendix Table S2: IHC and Western Blot Antibody details

Appendix Table S3: Flow Cytometry Antibody details

Appendix Figure S1 SERM-selective of suppression of IL11-mediated STAT3 signaling activity.

(A) Effect of *bazedoxifene* (BZA) and *tamoxifen* (TAM) on IL11-induced and STAT3-dependent pAPRE luciferase reporter activity in HEK293T cells expressing human IL11Rα. Cells were co-transfected with a non-responsive renilla-luciferase plasmid. Cultures were stimulated with IL11 (30 ng/ml) and the indicated concentrations of BZA or TAM for 18 h. Result are expressed as relative luciferase units (RLU), i.e. firefly luciferase activity normalized against renilla luciferase activity in each individual culture. Data is presented as fold change to values obtained for the IL11 alone treatment wells; Mean $\pm$ SEM, n=3 independent experiments, *p = 0.023, **p = 0.003, both values compared to IL11 treatment; ***p = 0.0001 compared to no treatment; 2-way repeated measures ANOVA, Tukey's multiple comparison test.

(B) Effect of *tamoxifen* (TAM) on the proliferation of IL11-stimulated (30 ng/ml) BAF/03 murine B-cell lines expressing express human IL11Rα, as determined by MTS-assay. Measurements were carried out 48 h after treatments. Data are mean $\pm$ SEM, n=3 independent experiments, ***p = 0.0001 compared to no treatment, ANOVA, Dunnett's comparison test.

Appendix Figure S2 Models of *bazedoxifene* interactions with Site III residues of the hexameric gp130 complex.

(A) Overlay of the two proposed *bazedoxifene* binding modes. The indole and azepanyl rings for each binding mode are indicated. *In silico* modeling identified two binding modes of *bazedoxifene* with gp130, shown in (C) and (E) as a grey ribbon with some Site III side-chains shown as sticks. *Bazedoxifene* is shown as purple or green sticks.

(B) Overlay of *tamoxifen* (orange sticks) with one of the *bazedoxifene* binding modes (purple sticks) via their common phenoxypropylamine core to illustrate the structural differences between the two compounds. When docked in this manner, *tamoxifen* is unable to bind to the gp130 Site III surface due to steric clashes with gp130 residues L3, P5, N92 and Y94. Additionally, the two methyl substituents on the *tamoxifen* amine are unable to reach the hydrophobic pocket in which the *bazedoxifene* azepanyl ring binds. In addition, there are differences in the types of interactions that *tamoxifen* could potentially

make with gp130. The *bazedoxifene* indole and phenol rings are able to interact with gp130 residues via hydrogen bonds, in contrast to the phenyl rings and ethyl group of tamoxifen which can not hydrogen bond. Similar steric clashes with gp130 residues are observed when *tamoxifen* is overlaid with the alternative *bazedoxifene* binding mode (shown as green sticks in (A)).

(C) The *bazedoxifene* indole ring hydroxyl substituent can form hydrogen bonds to Q78 and both the indole and phenol rings can form π - π interactions with Y94. L3 interacts with both the *bazedoxifene* indole ring phenol substituent and the pendant phenyl ring via hydrophobic interactions. The *bazedoxifene* azepanyl ring is located in a largely hydrophobic pocket surrounded by C6, C32, F36, I83 and Q91.

(D) Gp130 depicted as a grey molecular surface in the same view as in (C). The locations of W157 and L57 in IL6 (brown), which interact with gp130, illustrate how the *bazedoxifene* indole ring and azepanyl ring (green, purple) mimic W157 and L57, respectively.

(E) The *bazedoxifene* indole ring can form π - π interactions with Y94. The phenol substituent of *bazedoxifene* is able to form hydrogen bonds to N92. The *bazedoxifene* indole ring hydroxyl substituent can from hydrogen bonds with either the hydroxyl group of Y94 or the side-chain of E12. L3 can form hydrophobic interactions with the pendant phenyl substituent of *bazedoxifene*. The *bazedoxifene* azepanyl ring is located in the same pocket of gp130 as shown in (C).

(F) Gp130 depicted as a grey molecular surface in the same view as in (E). The locations of W157 and L57 in IL6 (brown), which interact with gp130, illustrate how the *bazedoxifene* indole ring and azepanyl ring (green, purple) mimic W157 and L57 interactions in this orientation.

Amino acid codes: asparagine (N), cysteine (C), glutamic acid (E), glutamine (Q), isoleucine (I), Leucine (L), phenylalanine (F), tyrosine (Y), tryptophan (W).

Appendix Figure S3 Identification of tumor-associated immune infiltrates by flow cytometry.

Flow cytometric analysis of immune cells isolated from colon tissue collected from wildtype mice, and colonic tumours derived from *Cdx2*$^{\mathrm{CreERT2}}$;*Apc*^{flox} mice treated with vehicle or *bazedoxifene* (BZA). Representative FACS plots showing the gating strategy to exclude

epithelial cells ($EpCAM^+$) and isolate immune cells ($EpCAM^-$,CD45+), T cells ($CD4^+$, $CD8^+$), and myeloid cell lineages with Ly6C, Ly6G, MHCII and F480 as markers.

Appendix Figure S4 Tumor infiltrating T-cells populations are unaffected in *bazedoxifene* (BZA) or vehicle-treated *Cdx2*^{CreERT2};*Apc*^{flox} mice.

Distribution of immune cell types identified by flow cytometry analysis in colon sections containing tumors from *Cdx2*^{CreERT2};*Apc*^{flox} mice treated with vehicle or BZA. Distal colon tissue of untreated wild-type mice (WT) were included as a reference.

(A) Representative flow cytometry plot showing $EpCAM^-$,$CD45^+$,$TCR\beta^+$,$CD8^+$ and $CD8^+$,$PD1^+$ T cell populations, and quantitation from three experimental cohorts.

(B) Representative flow cytometry plot showing $EpCAM^-$,$CD45^+$,$TCR\beta^+$,$CD4^+$ and $CD4^+$,$PD1^+$ T cell populations, and quantitation from three experimental cohorts.

Data are mean $\pm$ SEM, with n=3 mice per cohort, *p* value derived from unpaired T-test.

Appendix Figure S5 Tumor infiltrating myeloid and B cell populations are unaffected in *bazedoxifene* (BZA) or vehicle-treated *Cdx2*^{CreERT2};*Apc*^{flox} mice.

Distribution of immune cell types identified by flow cytometry analysis in colon sections containing tumors from *Cdx2*^{CreERT2};*Apc*^{flox} mice treated with vehicle or BZA. Distal colon tissue of untreated wild-type mice (WT) were included as a reference. Representative flow cytometry plots for specific immune cell subsets and quantitation in the three experimental cohorts are shown as follows:

(A) $EpCAM^-$,$CD45^+$,$Cd11b^+$,$Ly6G^{lo}$, $MHCII^+$, $F480^+$ macrophages,

(B) $EpCAM^-$,$CD45^+$,$Ly6C^{hi}$,$Ly6G^{lo}$ monocytic myeloid-derived suppressor cells (M-MDSCs) and $CD45^+$,$CD11b^+$,$Ly6C^{lo}$,$Ly6G^{hi}$ granulocytic MDSCs (G-MDSCs) populations, and

(C) $EpCAM^-$,$CD45^+$,$CD19^+$ B cell populations.

Data are mean $\pm$ SEM, with n=3 mice per cohort. Each symbol represents individual mice, *p* value derived from unpaired T-test.

A

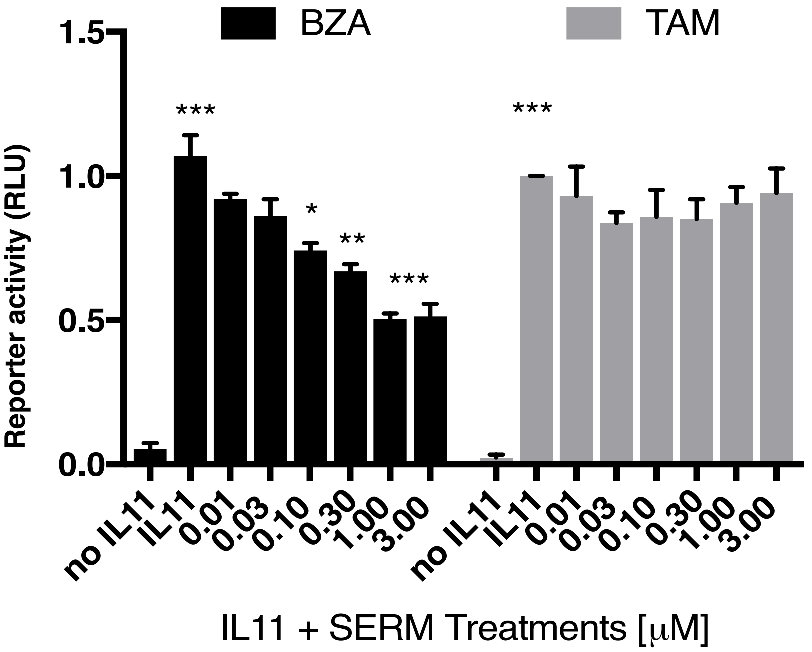
BZA
TAM
1.5
1.0
0.5
0.0
Reporter activity (RLU)
***
*
**
***
***
no IL11
IL11
0.01
0.03
0.10
0.30
1.00
3.00
IL11 + SERM Treatments [μM]

B

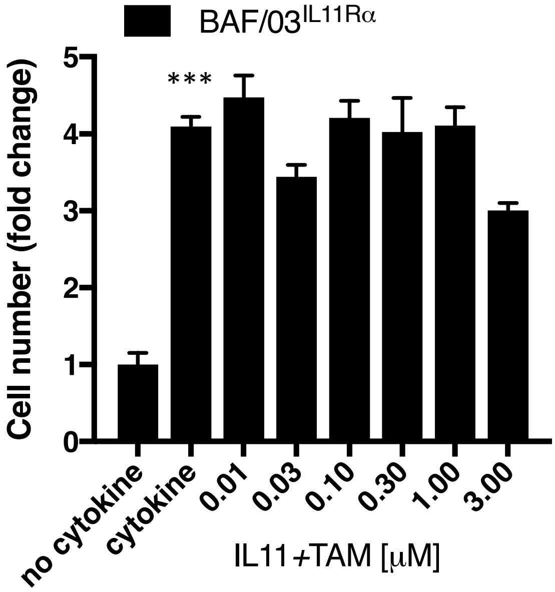
BAF/03IL11Rα
5
4
3
2
1
0
Cell number (fold change)
***
no cytokine
cytokine
0.01
0.03
0.10
0.30
1.00
3.00
IL11+TAM [μM]

Appendix Figure S2

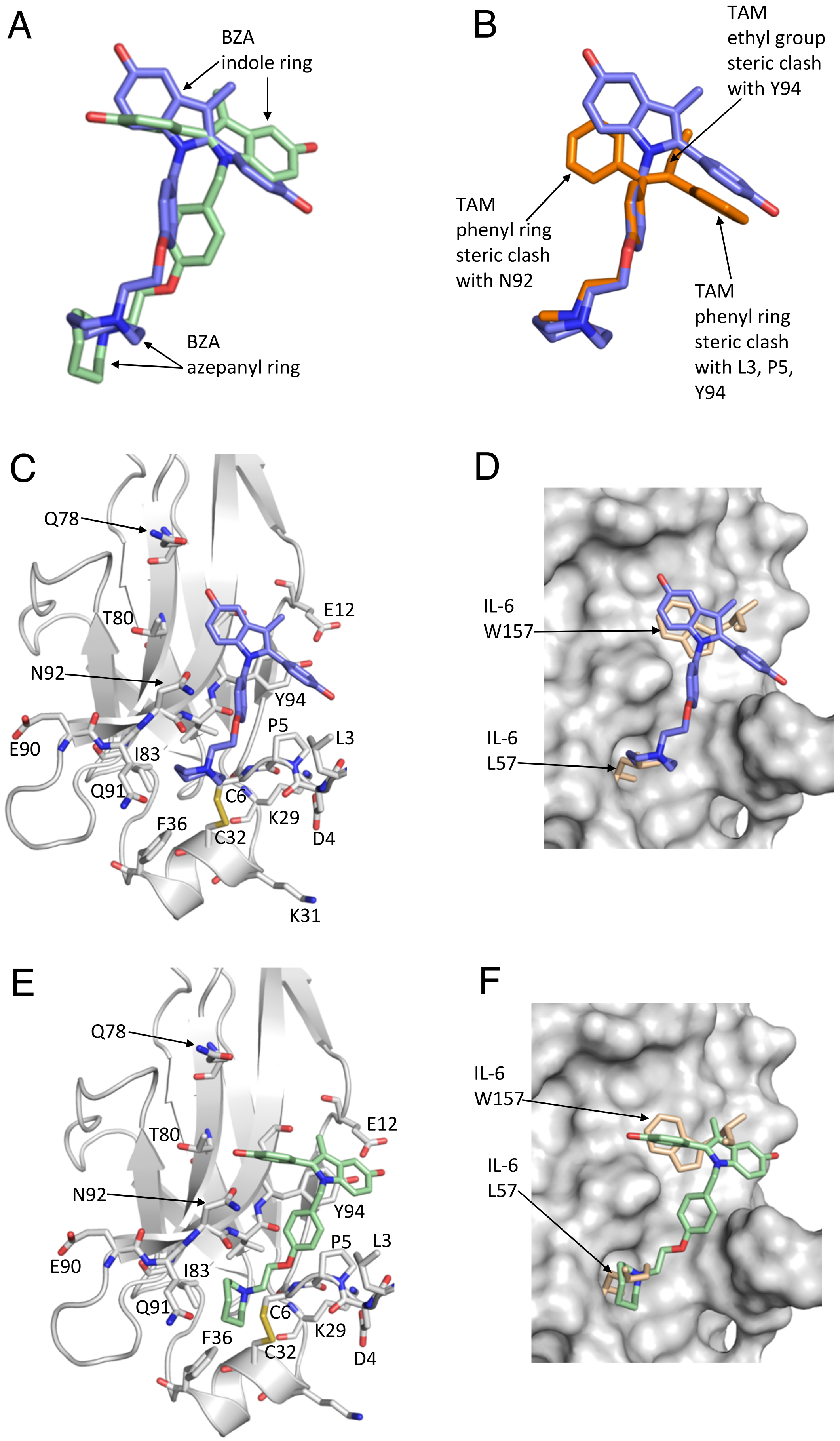

Appendix Figure S3

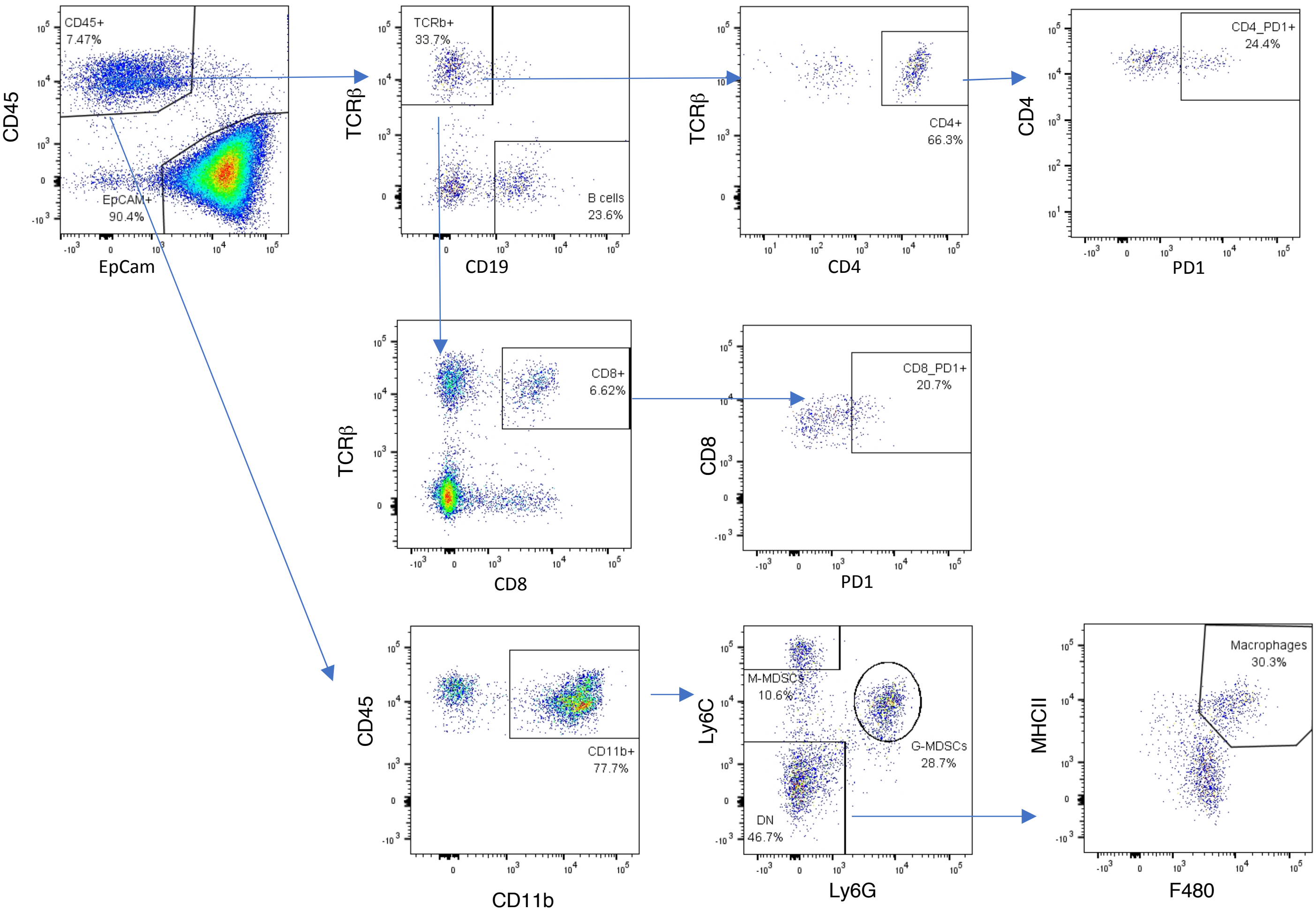

Appendix Figure S4

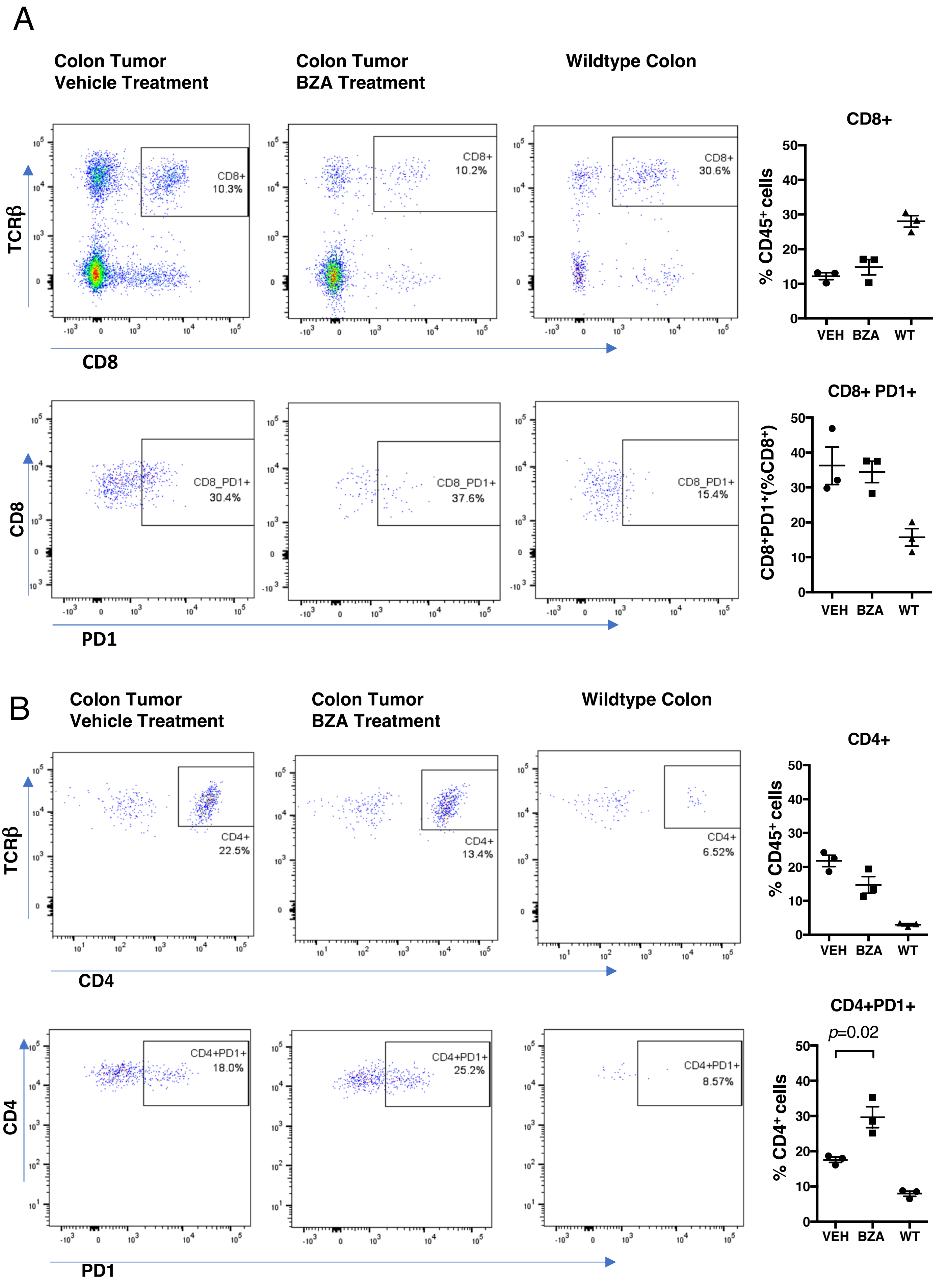

Appendix Figure S5

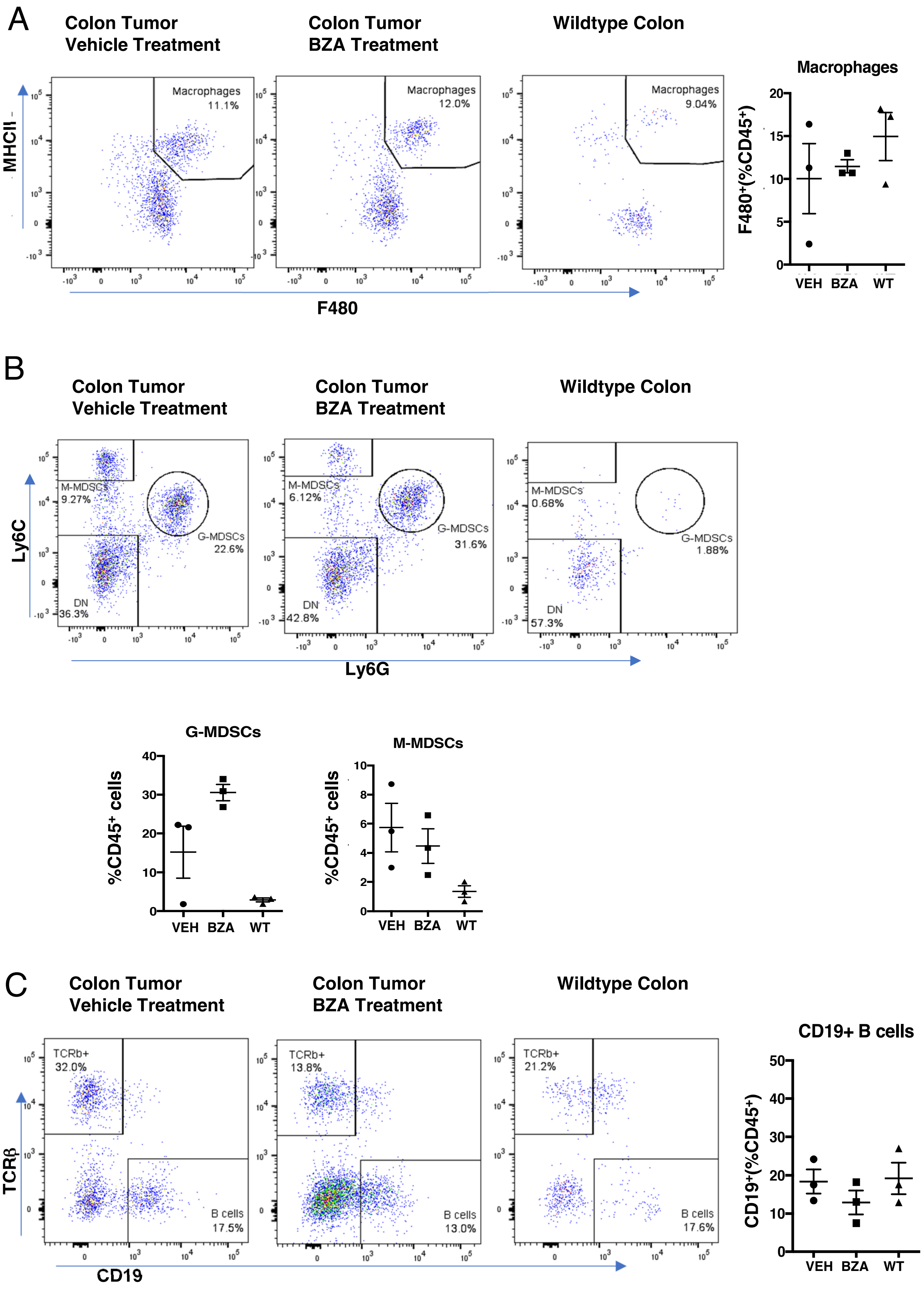

Appendix Table S1

**Human Primer Sequences for QPCR**	
**Gene**	**Primer Sequence**
HPRT Forward	CCCTGGCGTCGTGATTAGTG
HPRT Reverse	TGGGTCAGGGGTGGTTATTG
Il11 Forward	ATGAACTGTGTTTGCCGCCT
Il11 Reverse	GAATCCAGGTTGTGGTCCCC
IL6 Forward	CAATGAGGAGACTTGCCTGGTG
Il6 Reverse	TCTTGCCAGGTGACACTGAG
IL6ST Forward	AGATCCATCCCATACTCAAGGC
IL6ST Reverse	ACAGGGTGAGTAGCTTGAAAGTC

**Mouse Primer Sequences for QPCR**	
**Gene**	**Primer Sequence**
Hprt Forward	GGC CAG ACT TTG TTG GAT TTG
Hprt Reverse	CGC TCA TCT TAG GCT TTG TAT TTG
Il11 Forward	ATG AAC TGT GTT TGT CGC CTG
Il11 Reverse	CCA GGA AGC TGC AAA GAT CC
Il6 Forward	TCT ATA CCA CTT CAC AAG TCG GA
Il6 Reverse	GAA TTG CCA TTG CAC AAC TCT TT
Icam1 Forward	CAA TTT CTC ATG CCG CAC AG
Icam Reverse	AGC GGA AGA TCG AAA GTC CG
Reg3a Forward	CTGGTC TGC CAG AAG AGA CC
Reg3a Reverse	GGT AGT TGT CCA CTC TGC CG
Socs3 Forward	GCG GGC ACC TTT CTT ATC C
Socs3 Reverse	TCC CCG ACT GGG TCT TGA C
Axin2 Forward	AGC CTA AAG GTC TTA TGT GG
Axin2 Reverse	ATG GAA TCG TCG GTC AGT
Lgr5 Forward	CTT CAC TCG GTG CAG TGC T
Lgr5 Reverse	GAT CAG CCA GCT ACC AAA TAG G
Sox9 Forward	CCA CGG AAC AGA CTC ACA TCT CTC

Sox9 Reverse	CTG CTC AGT TCA CCG ATG TCC ACG
Gapdh Forward	CGA CCC CTT CAT TGA CCT TA
Gapdh Reverse	GCC TTG ACT GTG CTG TTG AA
Esr1 Forward	CCT CCC GCC TTC TAC AGG
Esr1 Reverse	CAC ACG GCA CAG TAG CGA

Appendix Table S2

**Antibodies used for Western Blot**			
**Antibody**	**Supplier**	**Catalog #**	**Dilutions Used**
Phospho-STAT3 (Tyr705)	Cell Signaling	#9145	1:500
STAT3	Cell Signaling	#4904	1:1000
Phospho-p44/42 MAPK (Erk1/2)	Cell Signaling	#4370	1:2000
p44/42 (Erk1/2)	Cell Signaling	#4695	1:1000
Akt	Cell Signaling	#9272	1:1000
Phospho-Akt	Cell Signaling	#4058	1:1000
BIM	Cell Signaling	#2819	1:1000
Cyclin D1	Cell Signaling	#2978	1:1000
MCL-1	Cell Signaling	#5453	1:1000
Survivin	Cell Signaling	#2808	1:1000
BCL-xL	BD Bioscience	#551022	1:1000
β-actin	Sigma	#A1978	1:1000
GAPDH	Sigma	#G8795	1:2000
**Antibodies used for Immunohistochemistry**			
Phospho-STAT3	Santa Cruz	SC-7993	1:150
Ki67	ThermoFisher	MAS14520	1:150
β-Catenin	BD Biosciences	#610153	1:200

Appendix Table S3

Antibodies used for Flow Cytometry				
**Marker**	**Label**	**Supplier**	**Catalog #**	**Dilutions Used**
EpCAM (CD326)	FITC	MACS	130102214	1:300
EpCAM (CD326)	APC	e-Bioscience	EBS-17-5791-80	1:300
TCR β	PE	BioLegend	109208	1:500
PD1	PF710	INVITROGEN	46998582	1:300
CD8a	PECy7	BD Pharmingen	561097	1:300
CD45.2	A700	WEHI		1:500
F4/80	FITC	MACS	130-102-327	1:300
CD11b	PE	BD Pharmingen	553311	1:400
Ly-6G	PECy7	BD Pharmingen	560601	1:300
Ly-6C	eF450	e-Bioscience	48-5932-82	1:50
