# Supplementary material for: Repurposing the selective estrogen receptor modulator bazedoxifene to suppress gastrointestinal cancer growth
Source: EMBO Mol Med. 2019 Mar 18;11(4):e9539. doi: 10.15252/emmm.201809539 (PMC6460354; doi:10.15252/emmm.201809539)
Supplement: Supplementary file 2 — Expanded View Figures PDF [file EMMM-11-e9539-s002.pdf]

Expanded View Figures

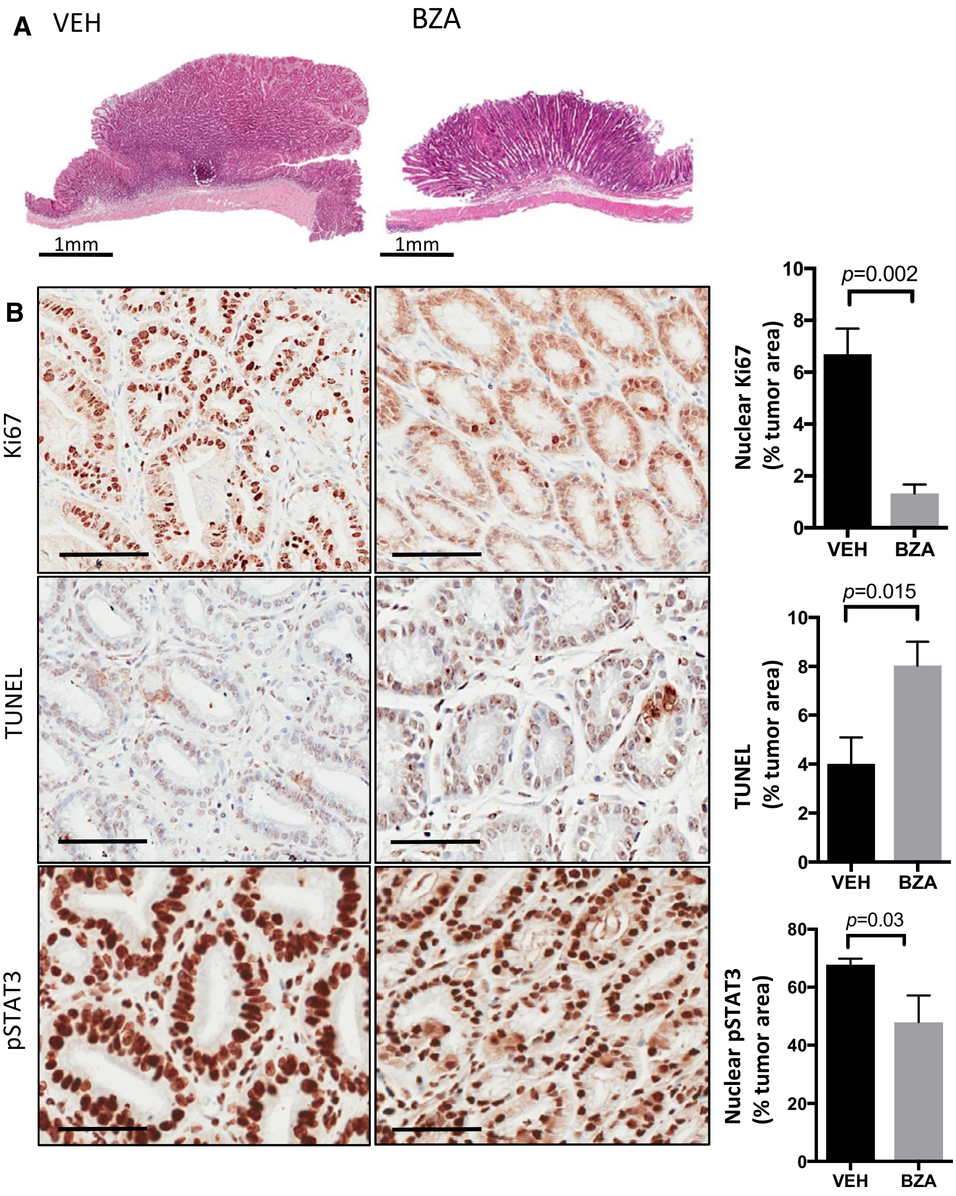

Figure EV1. *Bazedoxifene* treatment suppresses proliferation and induces apoptosis of gastric tumor epithelium in *gp130*^{Y757F} mice.

A Representative hematoxylin- and eosin-stained cross sections through the glandular stomach of 20-week-old *gp130*^{Y757F} mice that were treated with either *bazedoxifene* (BZA) or vehicle control (VEH) for 7 consecutive weeks. Scale bars = 1 mm.

B Representative immunohistochemistry staining for proliferative (Ki67 positive) and apoptotic (TUNEL positive) epithelium and those cells with high levels of the transcriptionally active pSTAT3 isoform (3+ intensity score) in sections of gastric tumors from mice as treated in (A). Immunohistochemical staining was quantified by Aperio analysis as outlined in the Materials and Methods section. Scale bars = 100 µm. Data are mean ± SEM, with n = 5 mice per cohort, P-value derived from unpaired Student's t test.

© 2019 The Authors

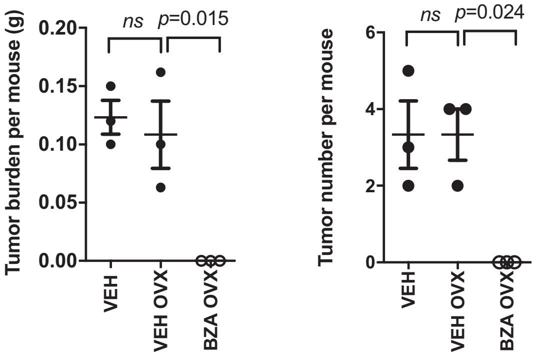

Figure EV2. *Bazedoxifene* reduces gastric tumor burden in the absence of circulating estrogens.

Total tumor burden and multiplicity determined in individual 20-week-old female mice that underwent either sham surgery or ovariectomy, and were subsequently treated with vehicle or *bazedoxifene* (BZA) for 7 weeks. Each symbol represents an individual mouse. Data are mean ± SEM, with $n = 3$ mice per cohort. ANOVA with Tukey's multiple comparisons test.

© 2019 The Authors

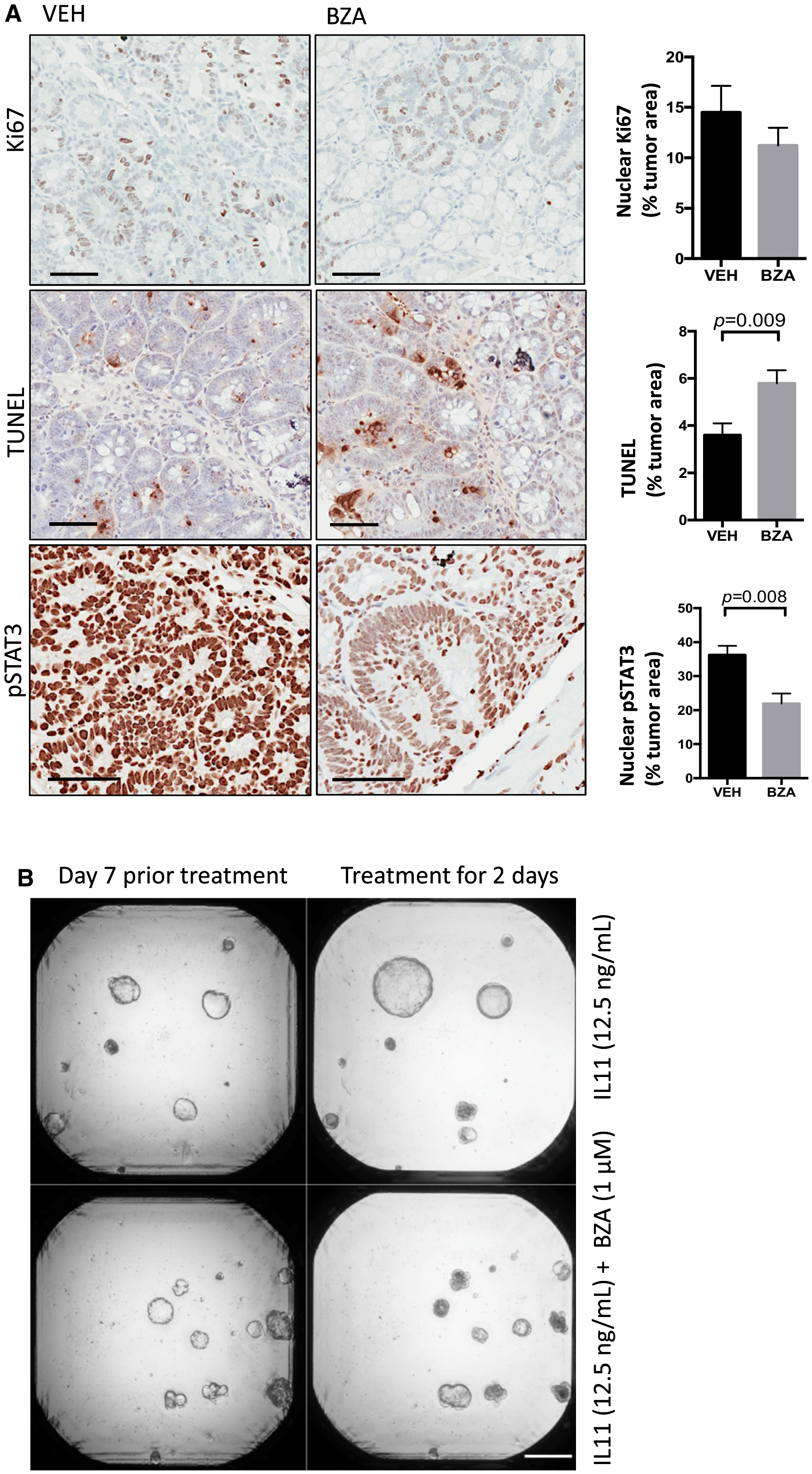

Figure EV3. *Bazedoxifene* treatment suppresses proliferation and induces apoptosis of colonic tumor epithelium in *Cdx2*^{CreERT2}; *Apc*^{flox} mice.

A Representative immunohistochemistry staining for proliferative (Ki67 positive) and apoptotic (TUNEL positive) epithelium and those cells with high levels of the transcriptionally active pSTAT3 isoform (3+ intensity score) in tumor sections of mice as treated in Fig 5A. Immunohistochemical staining was quantified by Aperio analysis as outlined in the Materials and Methods section. Scale bars = 100 μm. Data are mean ± SEM, with *n* = 5 mice per cohort, *P*-value derived from unpaired Student's *t* test.

B Representative images of colon cancer organoids derived from *Apc*^{Min} mice grown for 7 days in the presence of IL11 (12.5 ng/ml) alone or in combination with 1 μM *bazedoxifene* (BZA) for a further 2 days. Scale bar = 500 μm.

 © 2019 The Authors

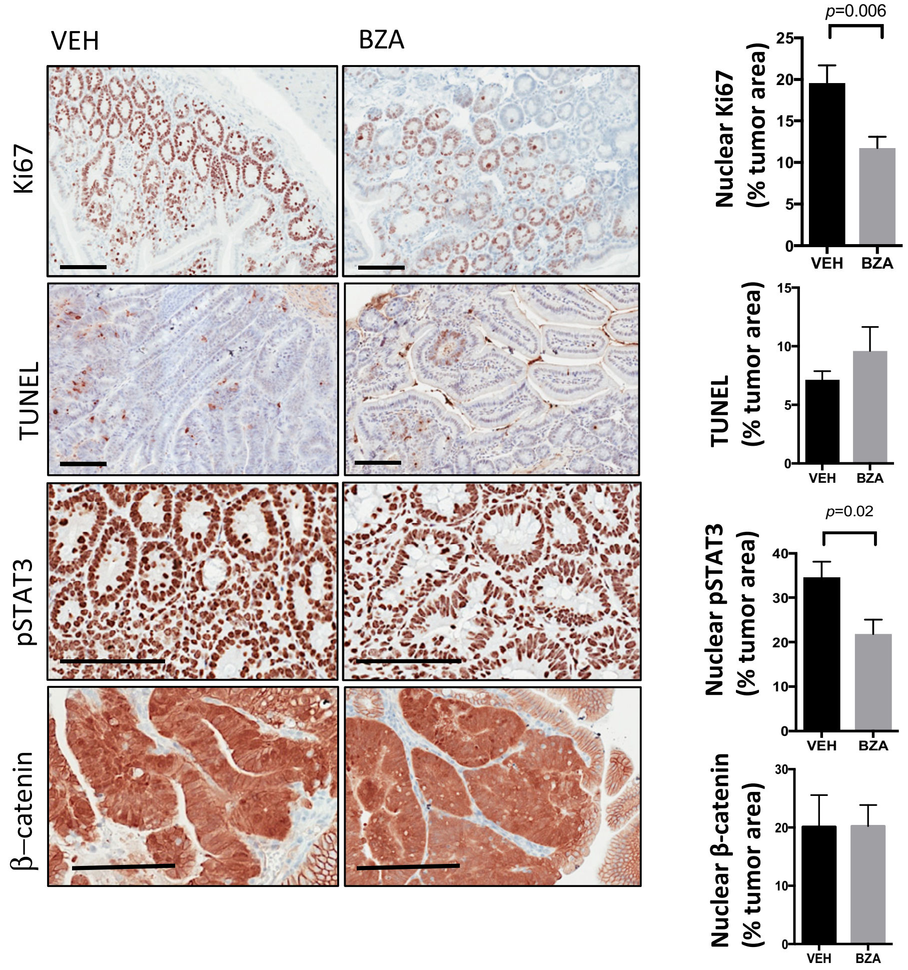

Figure EV4. *Bazedoxifene* treatment suppresses proliferation and induces apoptosis of tumor epithelium in the small intestine of *Lgr5*^{CreERT2}; *Apc*^{flox} mice independent of β-catenin signaling.

Representative immunohistochemistry staining for proliferative (Ki67 positive) and apoptotic (TUNEL positive) epithelium and those cells with high levels of the transcriptionally active pSTAT3 isoform (3+ intensity score) or β-catenin (nuclear accumulation) signaling in tumor sections of mice as treated in Fig 6A. Immunohistochemical staining was quantified by Aperio analysis as outlined in the Materials and Methods section. Scale bars = 100 μm. Data are mean ± SEM, with n = 5 mice per cohort, P-value derived from unpaired t test.

© 2019 The Authors
