# Supplementary material for: Repurposing the selective estrogen receptor modulator bazedoxifene to suppress gastrointestinal cancer growth
Source: EMBO Mol Med. 2019 Mar 18;11(4):e9539. doi: 10.15252/emmm.201809539 (PMC6460354; doi:10.15252/emmm.201809539)
Supplement: Supplementary file 4 — Source Data for Figure 3 [file EMMM-11-e9539-s003.pdf]

Source Data

Repurposing the selective estrogen receptor modulator *bazedoxifene* to suppress gastrointestinal cancer growth

Thilakasiri *et al*

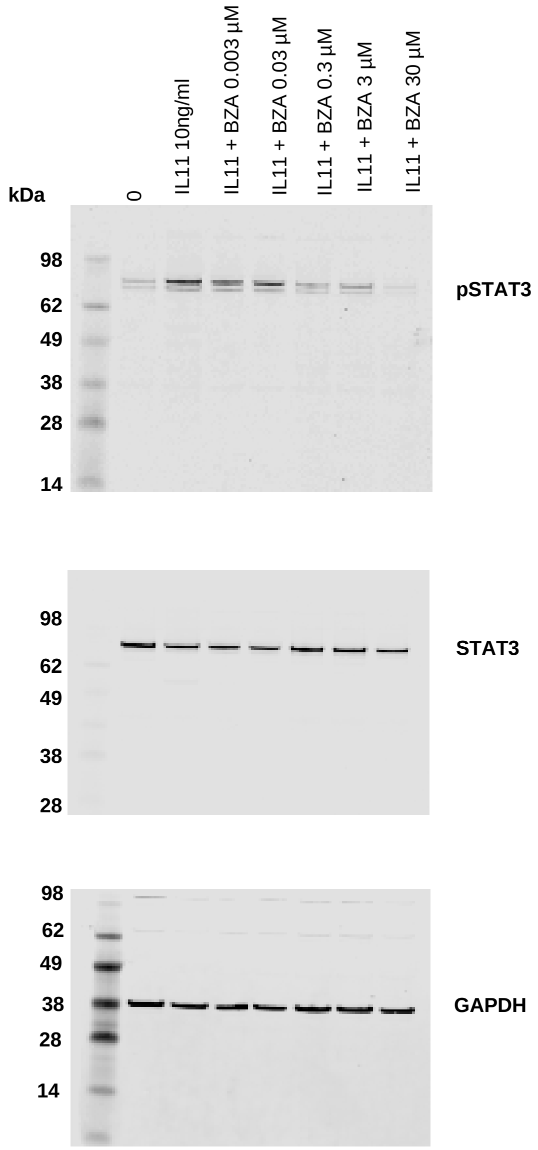

Thilakasiri *et al* **Figure 3A** IL11 stimulation of MKN1 cell line.

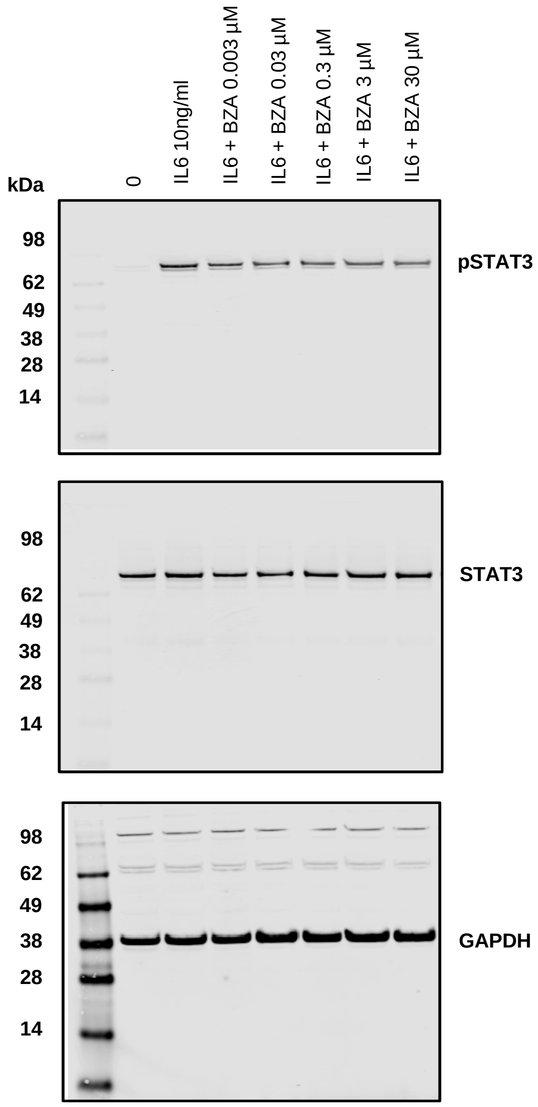

Thilakasiri *et al* **Figure 3A** IL6 stimulation of MKN1 cell line.

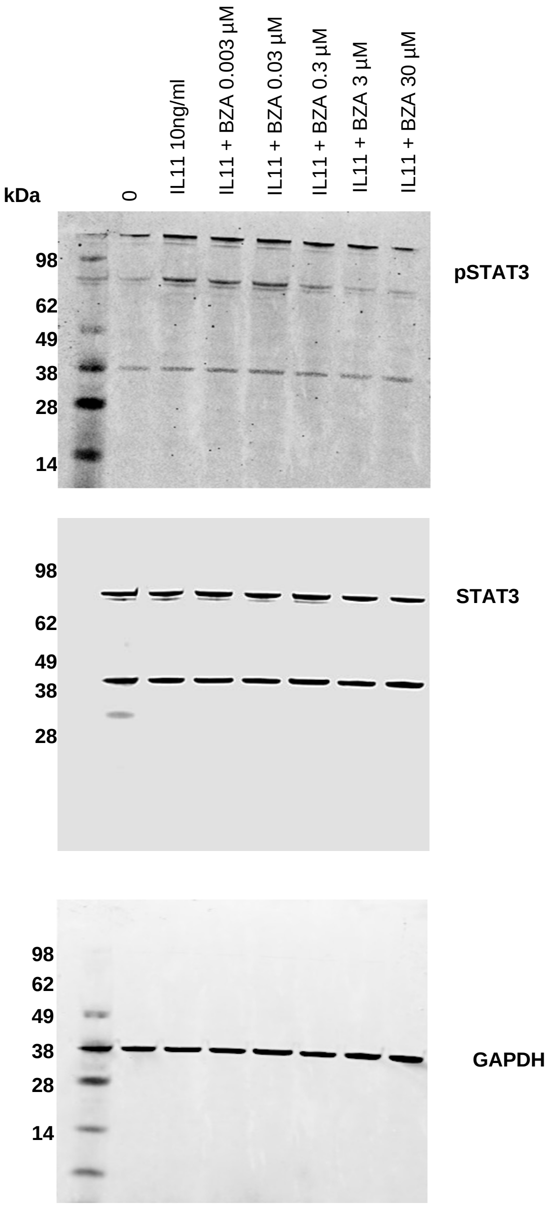

Thilakasiri *et al* **Figure 3A** IL11 stimulation of LIM2405 cell line.

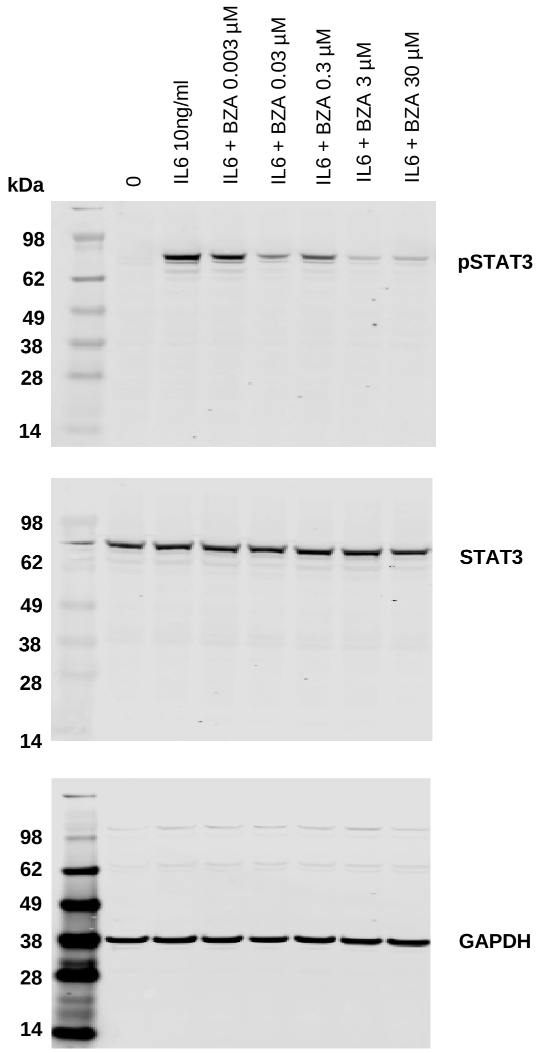

Thilakasiri *et al* **Figure 3A** IL6 stimulation of LIM2405 cell line.

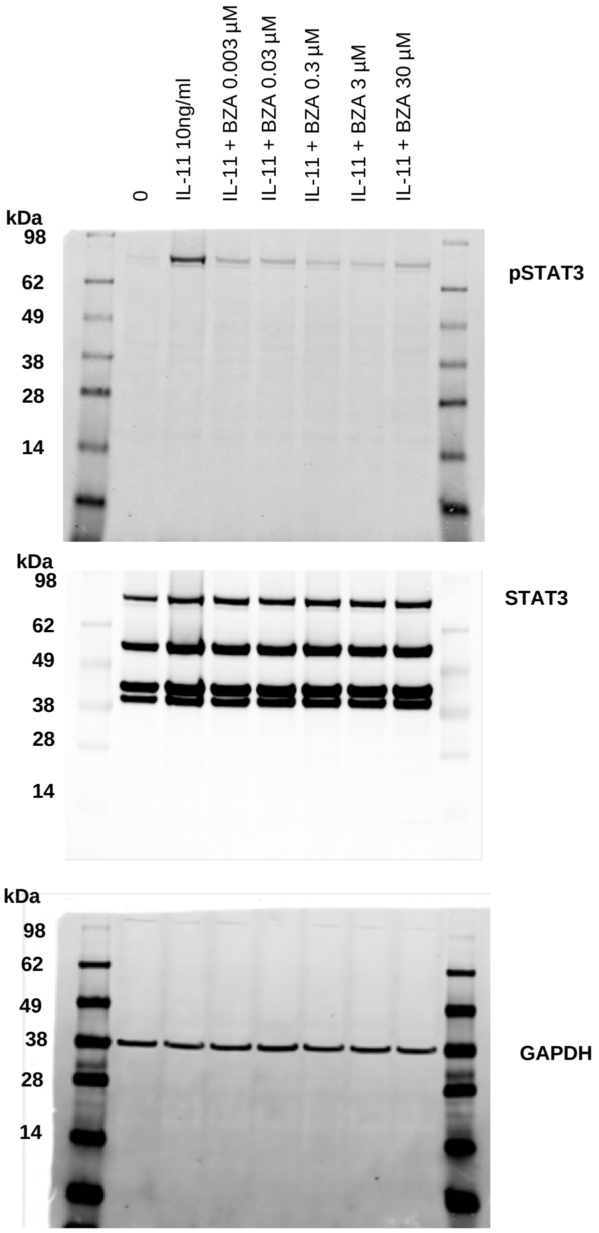

Thilakasiri *et al* **Figure 3A** IL11 stimulation of MDA-MB231 cell line.

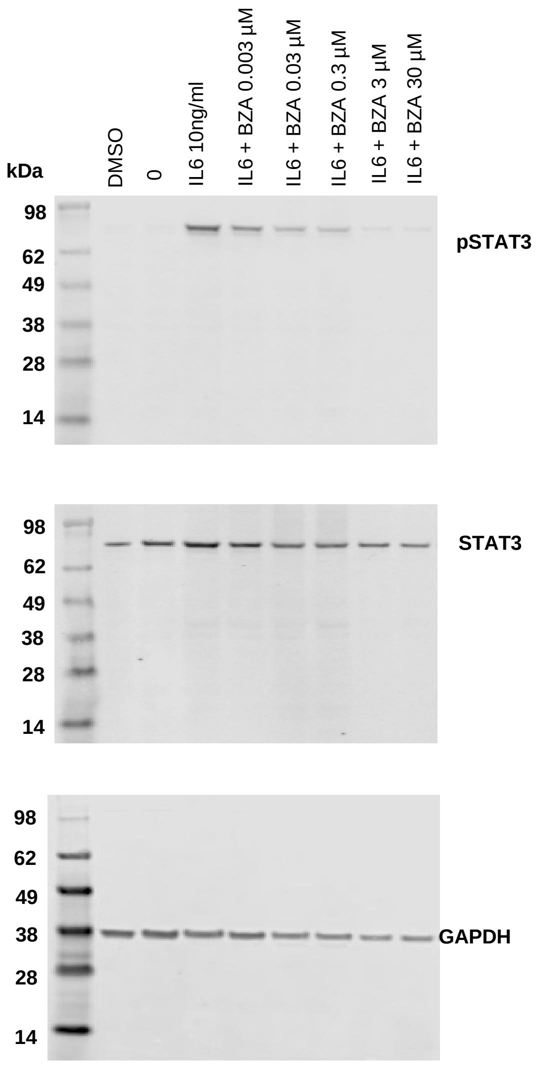

Thilakasiri *et al* **Figure 3A** IL6 stimulation of MDA-MB231 cell line.

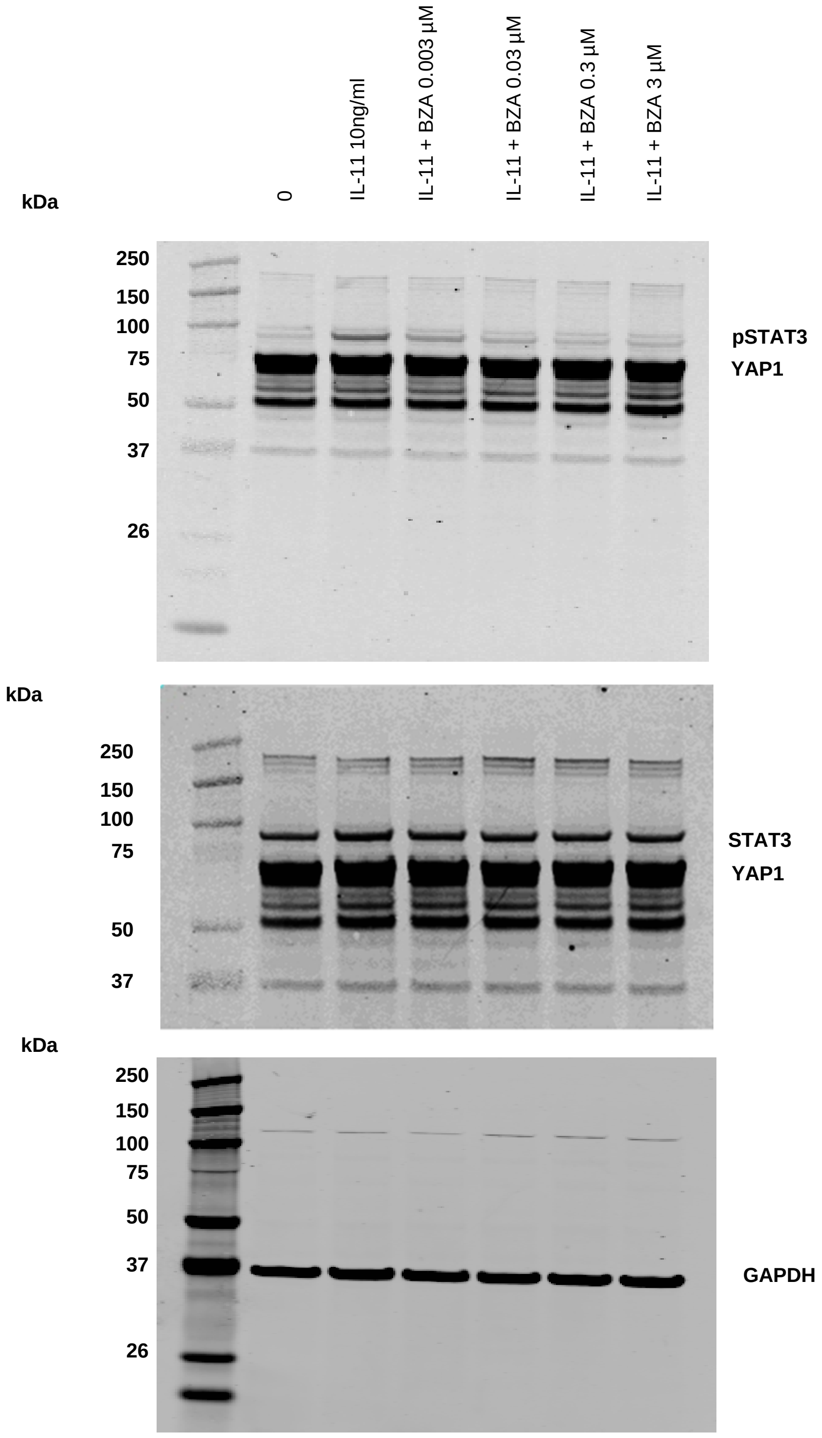

Thilakasiri *et al* **Figure 3B** IL11 stimulation of CRC1 cells.

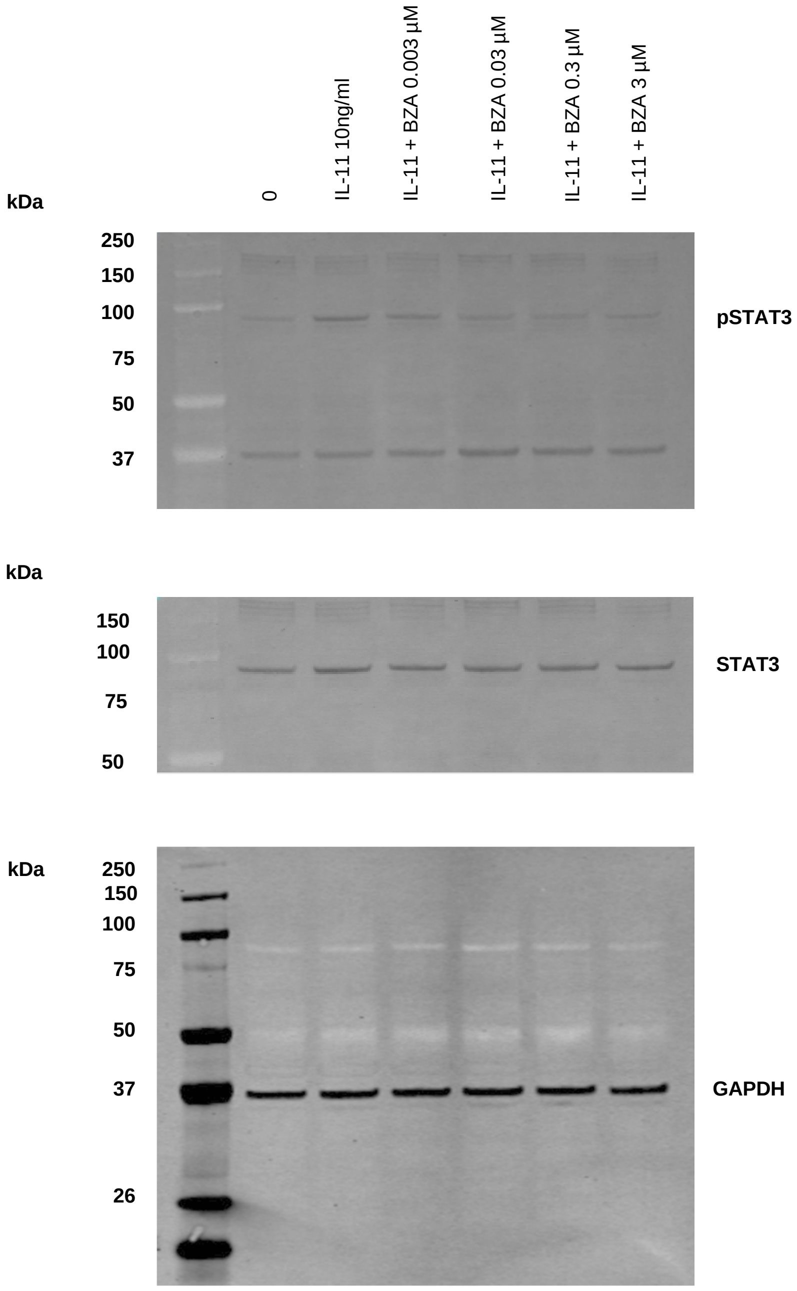

Thilakasiri *et al* **Figure 3B** IL11 stimulation of CRC2 cells.

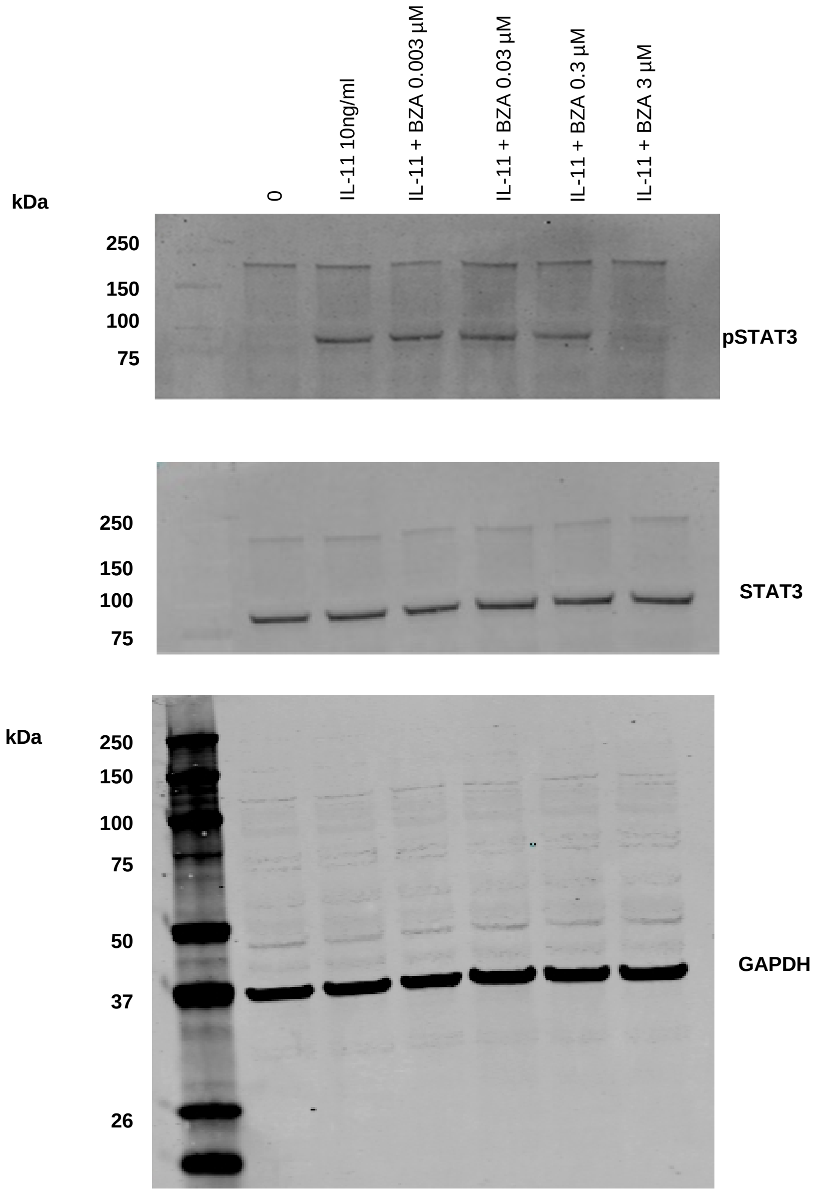

Thilakasiri *et al* **Figure 3B** IL11 stimulation of CRC3 cells.
