# Supplementary material for: Repurposing the selective estrogen receptor modulator bazedoxifene to suppress gastrointestinal cancer growth
Source: EMBO Mol Med. 2019 Mar 18;11(4):e9539. doi: 10.15252/emmm.201809539 (PMC6460354; doi:10.15252/emmm.201809539)
Supplement: Supplementary file 5 — Source Data for Figure 4 [file EMMM-11-e9539-s004.pdf]

Source Data

Repurposing the selective estrogen receptor modulator ***bazedoxifene*** to suppress gastrointestinal cancer growth

Thilakasiri *et al*

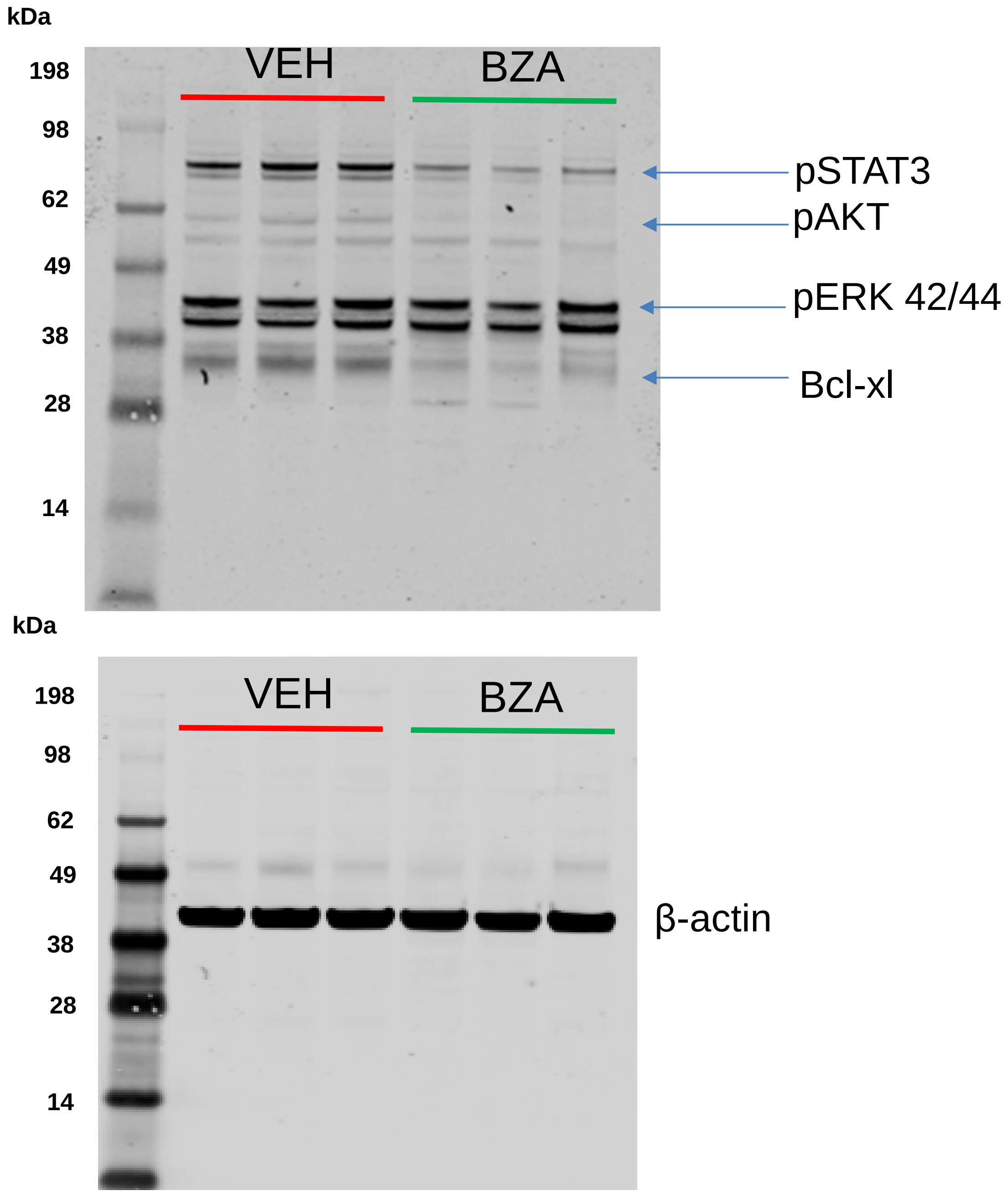

Thilakasiri *et al* **Figure 4F** Western Blot images of gp130^{Y757} tumour lysates

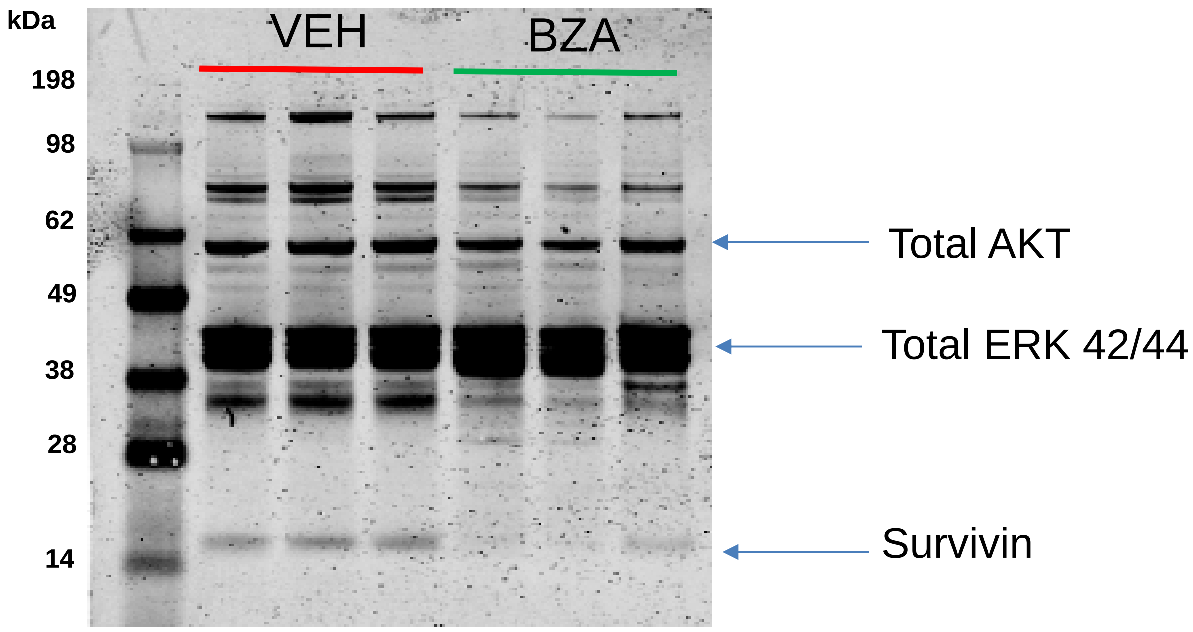

Thilakasiri *et al* **Figure 4F** Western Blot images of gp130^{Y757} tumour lysates

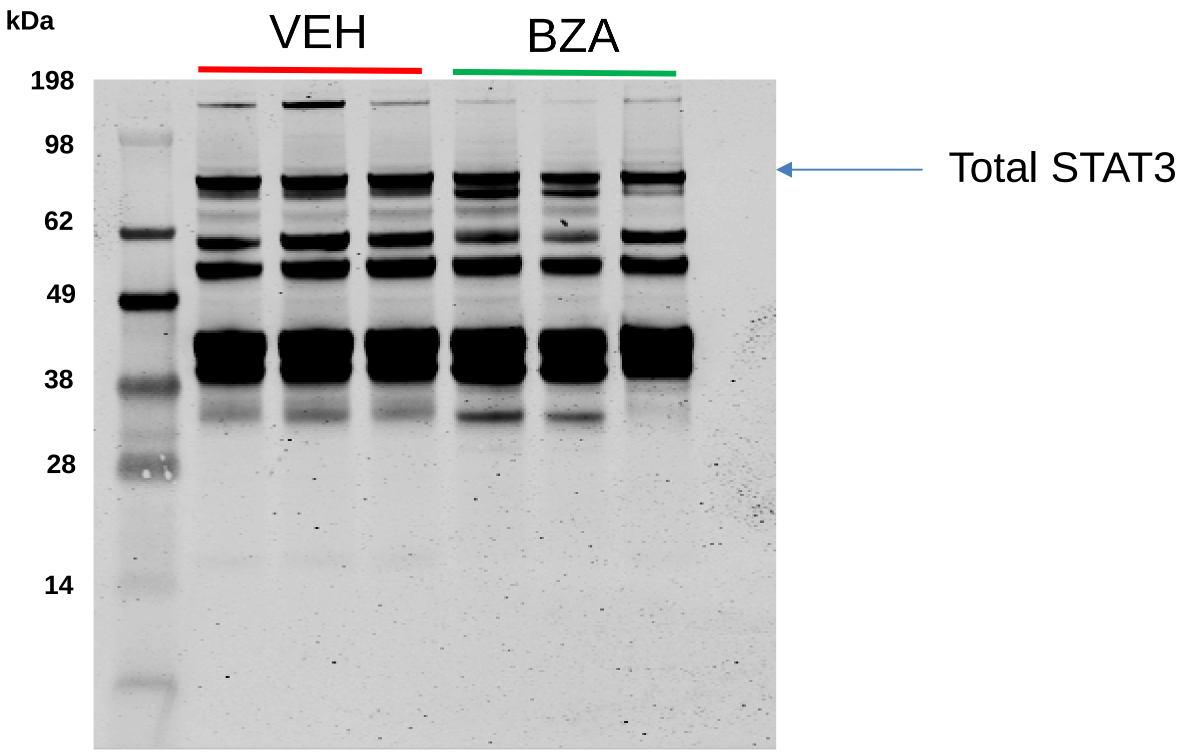

Thilakasiri *et al* **Figure 4F** Western Blot images of gp130^{Y757} tumour lysates
