# Supplementary material for: Repurposing the selective estrogen receptor modulator bazedoxifene to suppress gastrointestinal cancer growth
Source: EMBO Mol Med. 2019 Mar 18;11(4):e9539. doi: 10.15252/emmm.201809539 (PMC6460354; doi:10.15252/emmm.201809539)
Supplement: Supplementary file 6 — Source Data for Figure 5 [file EMMM-11-e9539-s005.pdf]

Source Data

Repurposing the selective estrogen receptor modulator *bazedoxifene* to suppress gastrointestinal cancer growth

Thilakasiri *et al*

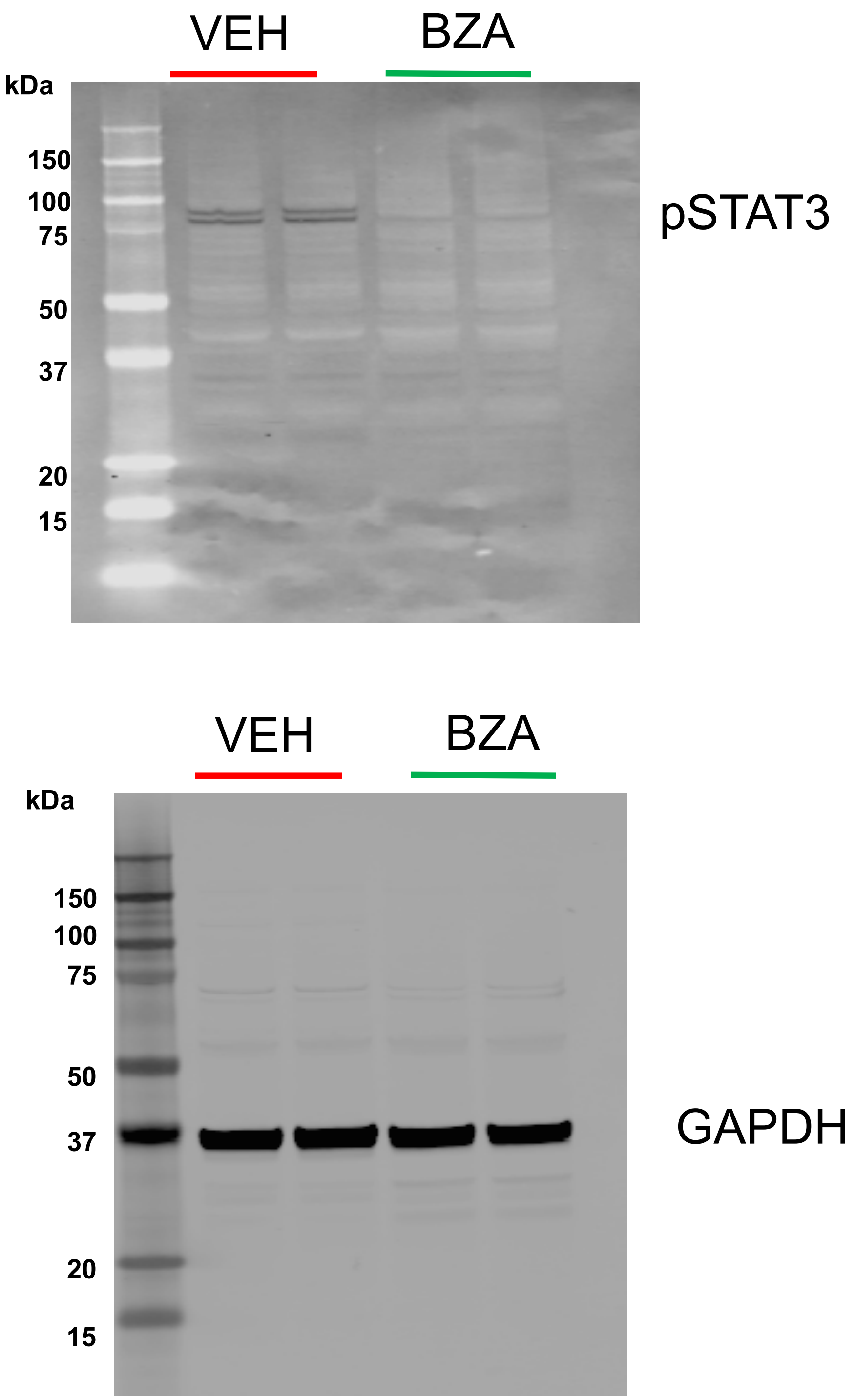

Thilakasiri *et al* **Figure 5G** Western blot images of Cdx2^{CRE}*APC*^{flox} tumour lysates

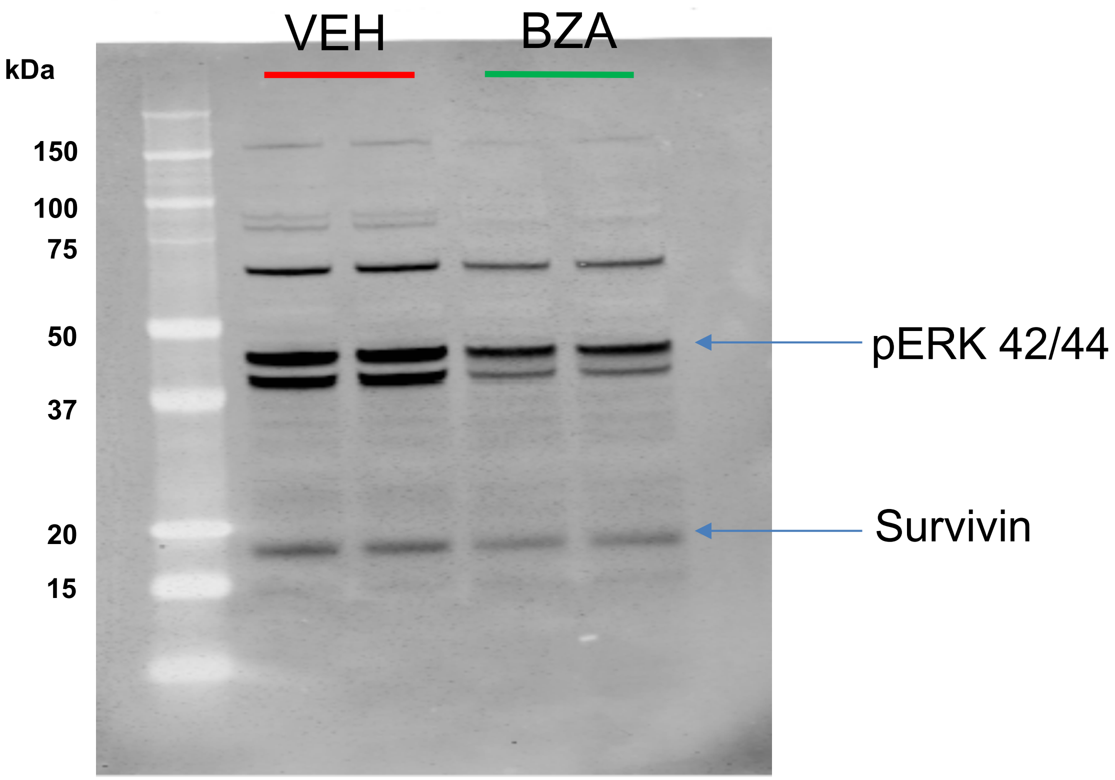

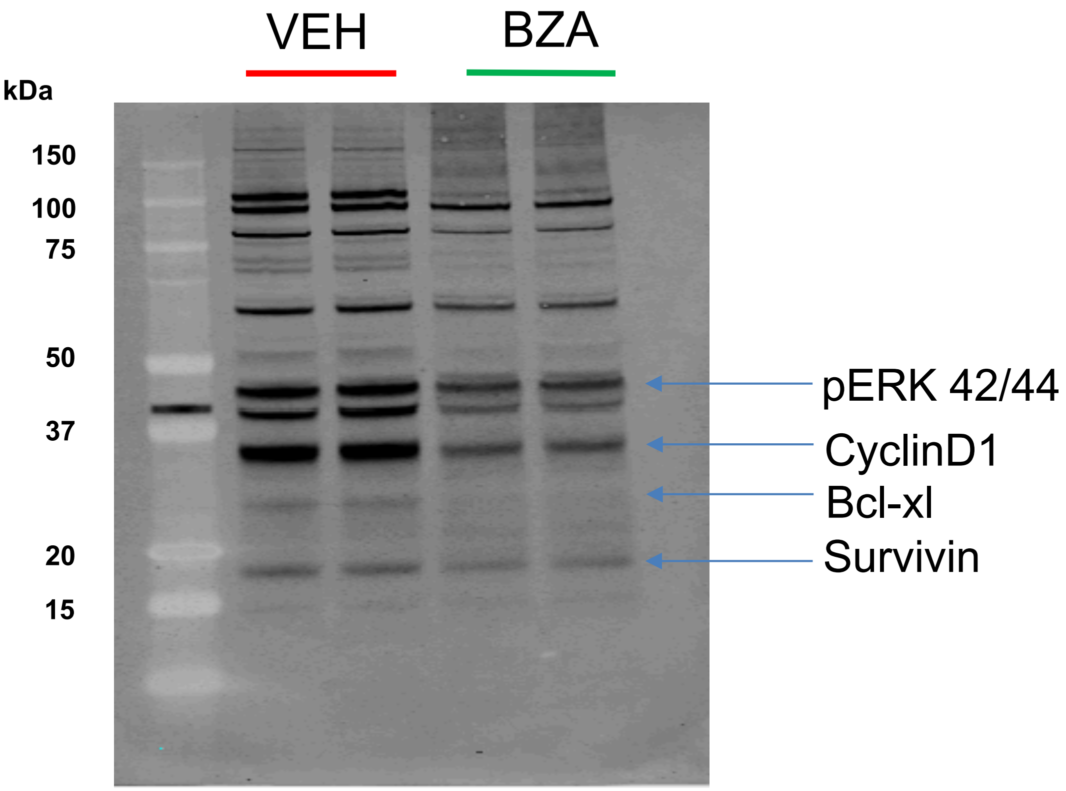

Thilakasiri *et al* **Figure 5G** Western blot images of Cdx2^{CRE}*APC*^{flox} tumour lysates

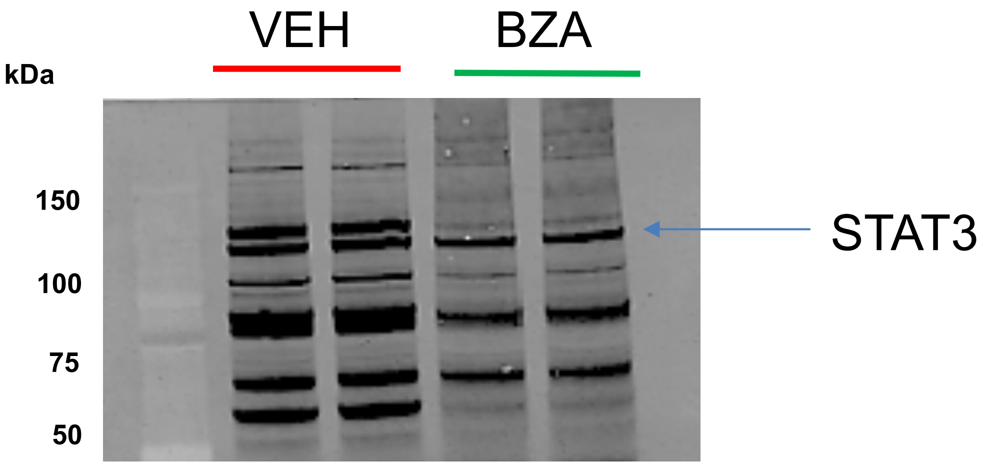

Thilakasiri *et al* **Figure 5G** Western blot images of Cdx2^{CRE}*APC*^{flox} tumour lysates
